# Rush Medical College

**Published:** 1861-02

**Authors:** 


### RUSH MEDICAL COLLEGE.

**NINETEENTH ANNUAL SESSION—1861-62.**

DANIEL BRAINARD, M. D., President,
*Professor of Surgery and Clinical Surgery.*

JAMES V. Z. BLANEY, M. D.,
*Professor of Chemistry and Pharmacy.*

J. ADAMS ALLEN, M. D.,
*Professor of Principles and Practice of Medicine, and Clinical Medicine.*

JOS. W. FREER, M. D.,
*Professor of Physiology, Microscopical and Surgical Anatomy.*

DE LASKIE MILLER, M. D.,
*Professor of Obstetrics and Diseases of Women and Children.*

EPHRAIM INGALS, M. D.,
*Professor of Materia Medica and Medical Jurisprudence.*

ROBERT L. REA, M. D., Secretary,
*Professor of Anatomy.*

EDWIN POWELL, M. D., Demonstrator.

The annual announcement will be issued soon.

☞ For information with regard to the institution, address the Secretary, R. L. REA, M. D.
*Chicago, Ill.*

